# Effect of *Serping1* siRNA Injection on Dopaminergic Cell Reduction in an MPTP-Induced Parkinson’s Disease Mouse Model

**DOI:** 10.3390/biomedicines14030569

**Published:** 2026-03-02

**Authors:** Min Hyung Seo, Sujung Yeo

**Affiliations:** 1 Department of Korean Medicine, Sang Ji University, Wonju 26339, Republic of Korea; cstcl@naver.com; 2 Research Institute of Korean Medicine, Sang Ji University, Wonju 26339, Republic of Korea

**Keywords:** Parkinson’s disease, *Serping1*, α-synuclein, substantia nigra, siRNA, MPTP

## Abstract

**Background**: Decreased dopaminergic cells and tyrosine hydroxylase (TH) in the substantia nigra (SN) lead to Parkinson’s disease (PD); but its cause remains unknown. PD is characterized by α-synuclein (α-syn) accumulation in Lewy bodies; most of which is phosphorylated at Ser129 (pSer129 α-syn). *Serping1* is an important gene for controlling blood vessel maintenance; including the process of inflammation. **Methods**: Increased expression of *Serping1* affects dopaminergic cell death in the SN of a chronic PD mouse model induced by 1-methyl-4-phenyl-1,2,3,6-tetrahydropyridine (MPTP); and *Serping1* siRNA treatment has a therapeutic effect in this model. **Results**: We demonstrated that this treatment shows a normal status in the motor ability test and TH level in the SN and striatum. *Serping1* siRNA was found to react to decreased *Serping1* levels in the SN. In the pSer129-α-syn level of the SN region; *Serping1* siRNA had a greater positive effect on PD than *N*-acetylcysteine by inhibiting pSer129-α-syn formation. Cyclooxygenase-2 and inducible nitric oxide synthase levels were decreased by *Serping1* siRNA treatment; thereby indicating its effect on inflammation. **Conclusions**: Our findings suggest that *Serping1* siRNA may represent a potential therapeutic approach for PD; warranting further investigation.

## 1. Introduction

Parkinson’s disease (PD) occurs as a result of decreased dopaminergic cells in the substantia nigra (SN), but the mechanism behind this decrease remains unknown. These cells play an important role in brain functions, including addiction, learning, reward seeking, eye movement, and motor planning. Drugs containing levodopa have been used for the effective treatment of PD, but long-term use of the drug results in levodopa-induced dyskinesia accompanied by chorea, dystonia, and hyperkinesis, including ataxia.

In previous studies, gene array analysis showed that the expression level of the serine/cysteine proteinase inhibitor (*Serping1*) gene increased in 1-methyl-4-phenyl-1,2,3,6-tetrahydropyridine (MPTP)-induced mouse models [1], and this increase has been associated with pathological changes in PD. *Serping1*, a complement component 1 inhibitor, is expressed in the tissues including the lungs, kidney, liver, blood, heart, and brain [2]. It is important for controlling a range of processes involved in blood vessel maintenance, including inflammation [3]. The analysis of gene expression in complement-related genes has identified *Serping1* upregulation in patients with pancreatic cancer [4] and in monocytes isolated from HIV+ patients [5]. An increased *Serping1* expression level has been reported to affect dopaminergic cell death in the SN of a chronic PD mouse model induced by MPTP [6]. Additionally, *Serping1* small interfering RNA (siRNA) treatment increases TH expression and decreases α-synuclein (α-syn) expression in SH-SY5Y cells, suggesting positive effects on pathological changes in PD [6]. These previous studies led to the idea that *Serping1* siRNA could be applied to PD for treatment.

Used as a tool to study single gene function in vivo and in vitro, siRNAs are attracting attention as potential therapeutics because of their ability to repress the function of specific genes [7]. Currently, siRNAs are suggested as an attractive new class of therapeutics for cancer and other diseases. Since Patisiran was approved as the first RNA interference therapeutic by the U.S. Food and Drug Administration in 2018, siRNA has been considered a promising therapeutic. Therefore, *Serping1* siRNA was applied to the MPTP-induced PD mouse model for PD therapy in this study to examine its therapeutic effects.

In patients diagnosed with PD, α-syn accumulates in Lewy bodies in the brain, and these bodies are composed of modified forms of α-syn, including phosphorylation, nitration, and ubiquitination in PD. In particular, most of the accumulated α-syn is phosphorylated at residue Ser129 (pSer129 α-syn), and postmortem analysis revealed that oligomeric pSer129 α-syn levels increased in patients with PD [8,9]. Given that pSer129 α-syn is considered a biomarker of PD today [10,11], pSer129 α-syn may be critical in PD diagnosis. In this study, the changes in pSer129 α-syn in the SN region were examined according to the *Serping1* siRNA treatment.

*N*-acetylcysteine (NAC), an acetylated cysteine compound, is being regarded as a potential therapeutic candidate for a broad spectrum of vascular and non-vascular neuropathies and neurodegenerative conditions, including PD [12]. In this study, NAC and *Serping1* siRNA differ substantially in their mechanisms of action; NAC exerts broad antioxidant and systemic effects, whereas *Serping1* siRNA specifically targets the inflammatory–complement pathway. Furthermore, different delivery vehicles were used for NAC and siRNA administration. Despite these mechanistic differences, NAC was used as a positive control to compare the degree of alleviation of PD pathology with that of *Serping1* siRNA.

The aim of this study was to verify that *Serping1* siRNA could be applied to PD for treatment based on the previous study. The primary hypothesis of this study was that the siRNA-mediated knockdown of *Serping1* would alleviate PD pathology in an MPTP-induced model. Our results showed that *Serping1* siRNA maintained the TH levels in the SN and striatum regions and inhibited pSer129-α-syn formation in SN and an inflammatory reaction in ST, supporting the assumption that in this model, siRNA could be explored as a potential treatment candidate for PD.

## 2. Materials and Methods

### 2.1. PD Induced by MPTP

**Figure 1:**
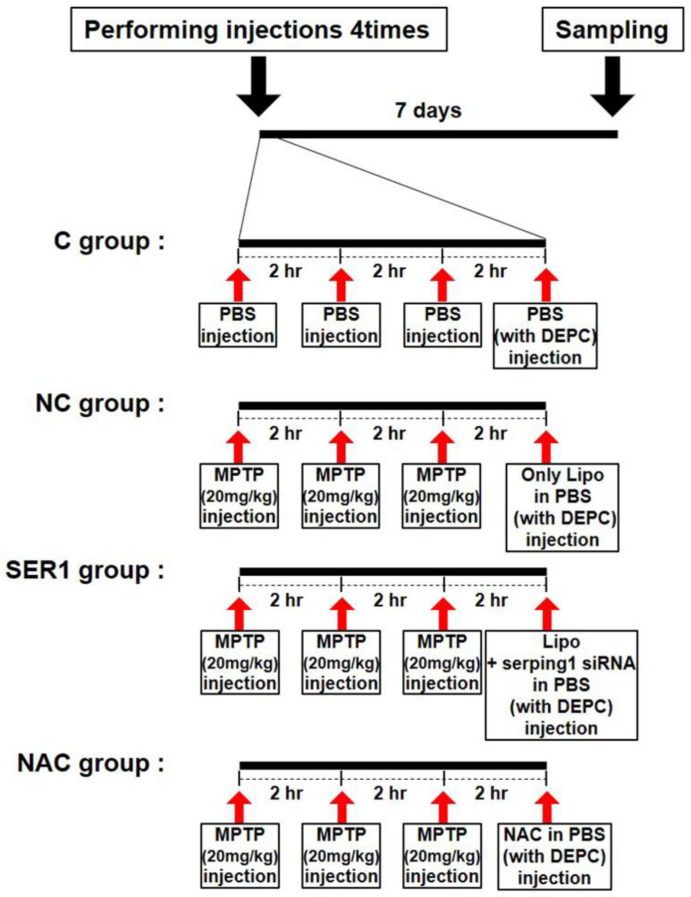
Brief description of experimental procedures in vivo. Eleven-week-old male inbred C57BL/6J mice were injected four times intraperitoneally on the first day. In the control (C) group, phosphate-buffered saline (PBS) was injected three times with an interval of 2 h instead of MPTP-HCL injections, and after 2 h, PBS made of diethylpyrocarbonate (DEPC) water was injected in a volume of 100 μL. In the negative control (NC) group, MPTP-HCL (20 mg/kg) was injected three times with an interval of 2 h, and after 2 h, only lipofectamine in PBS with DEPC was injected in a volume of 100 μL. In the *Serping1* (SER1) group, MPTP-HCL (20 mg/kg) was injected in the same way as in the NC group, and thereafter, lipofectamine with *Serping1* siRNA (5 mg/kg) in PBS with DEPC was injected in a volume of 100 μL. In the *N*-acetylcysteine (NAC) group, MPTP-HCL (20 mg/kg) was injected three times every 2 h, and after 2 h, NAC (150 mg/kg) in PBS with DEPC was injected in a volume of 100 μL.

Eleven-week-old C57BL/6J male mice (*n* = 6/a group; 25–27 g; DBL, Daejeon, Republic of Korea) were used in this study. They were divided into four groups: control (C), negative control (NC), *Serping1* siRNA injection (SER1), and *N*-acetylcysteine (NAC). To induce PD, mice were given three intraperitoneal injections of MPTP-HCl (20 mg/kg, free base) in PBS at 2-h intervals in the NC, SER1, and NAC groups before injecting therapeutic materials. By contrast, PBS instead of MPTP-HCL was injected three times at an interval of 2 h in the control group. Therapeutic materials for testing were injected intraperitoneally 2 h after the third injection. Sampling was performed 7 days after the first injection (Figure 1) with mice anesthetized using Alfaxan (Zoetis, Parsippany, NJ, USA). Transcardial perfusion was also conducted with cold PBS for Western blotting. All animal experiments in this study were approved by the Institutional Animal Care and Use Committee (IACUC) of Sang Ji University (IACUC protocol approval #2021–8) on 5 March 2021.

### 2.2. Preparation of the Therapeutic Materials

In the SER1 group, 1 mM *Serping1* siRNA (Bioneer, Daejeon, Republic of Korea) and lipofectamine reagent (Invitrogen, Carlsbad, CA, USA) were diluted in PBS with diethylpyrocarbonate (DEPC), where the *Serping1* siRNA (5′-CAC CUA UGU GAA UGC AUC U-3′) was purchased from Bioneer Inc., Daejeon, Republic of Korea. The diluted *Serping1* siRNA duplex was combined with diluted lipofectamine and incubated for 20 min at 21 °C. *Serping1* siRNA (5 mg/kg; mouse weight) and lipofectamine (2.3%) were injected in each mouse. Lipid nanoparticles (LNPs) and cationic lipid formulations, such as Lipofectamine, protect siRNA from nuclease degradation, thereby markedly increasing its circulation stability and in vivo activity [13,14,15]. Duplex siRNAs are thermodynamically stable at 37 °C [14,15].

In the NC group, only lipofectamine reagent was diluted in PBS with DEPC and incubated for 20 min at 21 °C in a manner similar to the treatment of the SER1 group, and lipofectamine (2.3%) was injected at 100 μL per mouse. In the control group, only PBS with DEPC (100 μL) was injected into each mouse, whereas in the NAC group, NAC (150 mg/kg) in PBS with DEPC (100 μL) was injected in each mouse. As NAC, a thiol-containing compound that functions as a redox modulator and precursor for glutathione synthesis, is known to decrease cellular damage by free radicals [16], NAC treatment was performed to build a positive control for comparison with the SER1 group.

When manufacturing therapeutic materials, including dialkyl indocarbocyanine (DiI), a stock solution was prepared by mixing CM-DiI (Invitrogen) and ethanol in a ratio of 1:1. Next, 0.5 μL of stock solution was added to 100 μL of the therapeutic materials in each group.

### 2.3. Motor Ability Test

**Figure 2:**
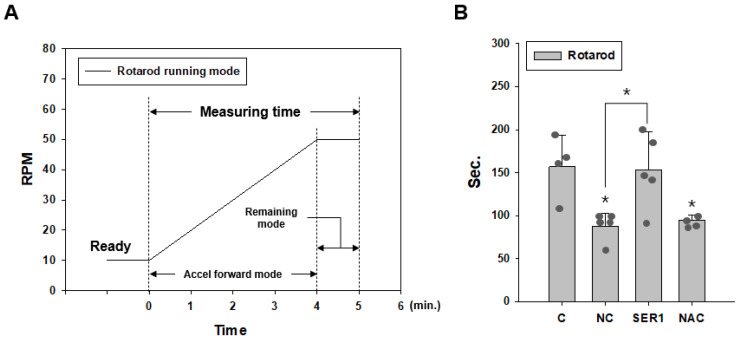
Rotarod test for evaluating motor ability in each group. (**A**) The motor ability test using a rotarod with a diameter of 28 mm was performed in Accel Forward mode for 4 min from 10 to 50 rpm during 5 min of running time, remaining at 50 rpm during the final minute. (**B**) The motor ability of the NC and NAC groups decreased significantly. However, that of the SER1 group was maintained at the control group (C) level; (control and NAC, *n* = 4; NC and SER1, *n* = 5; F(3,14) = 4.162, ANOVA = 0.024), * *p* < 0.05.

To evaluate motor ability in all groups, the rotarod test using a rotarod with a diameter of 28 mm was performed in Accel Forward mode for 4 min from 10 to 50 rpm during 5 min of running time, remaining at 50 rpm during the last minute (Figure 2A). Time was measured from the start to the first fall or first drop. The motor ability evaluation test was performed on day 6.

### 2.4. Western Blotting

The bilateral striatum and SN regions were extracted from the brains of mice perfused with cold PBS, and the tissues were homogenized in radioimmunoprecipitation assay buffer (20 mM) for 30 min on ice. After being centrifuged at 13,572× *g* at 4 °C for 20 min, the supernatant of the samples was quantified by bicinchoninic acid assay and separated by electrophoresis. Separated proteins were transferred to polyvinylidene difluoride membranes (Pall Corp., Port Washington, NY, USA), and the membranes were blocked with 3% bovine serum albumin (GenDEPOT, Baker, TX, USA) for an hour and a half at room temperature. Then, the membranes were incubated with primary antibodies: anti-TH (1:2000; Santa Cruz Biotechnology, Dallas, TX, USA), anti-*Serping1* (1:2000; Cloud clone Corp., Katy, TX, USA), anti-β-actin (1:5000; Santa Cruz Biotechnology), anti-pSer129-α-syn (1:1000; FUJIFILM Wako Pure Chemical Corp., Osaka, Japan), anti-cyclooxygenase-2 (COX2) (1:2000; Proteintech group, Rosemont, IL, USA), and anti-nitric oxide synthase (iNOS) (1:2000; Calbiochem, Darmstadt, Germany). Thereafter, the membranes were washed with Tris-buffered saline (pH 7.4) containing 0.1% Tween-20 (TBST) and incubated with the appropriate secondary antibodies: anti-mouse (1:5000; Abcam, Cambridge, UK) or anti-rabbit (1:5000; Abcam; Cambridge, MA, USA) IgG. The antigen–antibody complexes were visualized using Alliance Q9 Micro (UVITEC, Cambridge, UK) equipment for chemiluminescence imaging.

### 2.5. Immunohistochemistry

The brains of mice were resected and fixed in 4% paraformaldehyde for 18 h at 4 °C, and then washed with cold PBS and dehydrated with sucrose for 24 h at 4 °C. Cryosectioned brains were cut using a cryomicrotome in the coronal direction (30 μm thickness). The striatum and SN sections were washed with cold PBS three times for 5 min and incubated in 3% H_2_O_2_ in PBS (pH 7.4) for 15 min. After incubation with bovine serum albumin (3%) and Triton X-100 (0.3%) in PBS for an hour, the striatum and SN sections were blocked with a blocking buffer (1% bovine serum albumin, 5% horse serum in PBS) for an hour at room temperature prior to incubation with anti-TH antibody (1:2000; Santa Cruz Biotechnology) or anti-pSer129-α-syn (1:1000; FUJIFILM Wako Pure Chemical Corp.) as the primary antibody. The sections were treated with a biotinylated anti-mouse IgG and an avidin-biotin-peroxidase complex using a mouse immunodetection kit (Vector Laboratories, Newark, CA, USA) and an ABC kit (Vector Laboratories). Stained TH was developed using diaminobenzidine (0.003% 3,3-diaminobenzidine) hydrogen peroxide (0.03% in 0.05 M-Tris) solution (pH 7.0).

### 2.6. Immunofluorescence

The striatum sections obtained from cryosectioned brains were washed with cold PBS three times for 5 min each and incubated in 0.3% Triton X-100 for 30 min. After incubation in blocking buffer (1% bovine serum albumin, 5% goat serum in PBS) for an hour, the striatum sections were incubated with the primary antibodies, mouse anti-TH (1:1000; Santa Cruz Biotechnology) and rabbit anti-COX2 (1:2000; Proteintech group). Then, secondary antibodies, goat anti-mouse IgG(H+L) fluorescein isothiocyanate (FITC)-conjugated (CUSABIO, Houston, TX, USA) and goat anti-rabbit IgG (H+L) tetramethylrhodamine (TRITC)-conjugated (Novex, Waltham, MA, USA), were used. The striatum sections were treated with 4′,6-diamidino-2-phenylindole (DAPI; 1 μg/mL). Photographic documentation was conducted using a Nikon X-cite series 120Q microscope (Nikon, Tokyo, Japan).

To observe DiI, obtained from cryosectioned brains in immunohistochemistry assay, SN sections were washed with cold PBS three times for 5 min and treated with DAPI (1 μg/mL). After washing DAPI three times for 10 min each, the SN area was observed using a Nikon X-cite series 120Q microscope.

### 2.7. Imaging Software

ImageJ software (Version 1.52a) developed by the National Institutes of Health and the Laboratory for Optical and Computational Instrumentation was used for adjusting Western blotting images and microscopic images. SigmaPlot software (Version 12.5) was used for graph images.

### 2.8. Statistical Analysis

Student’s *t*-test and analysis of variance (ANOVA) in SPSS 25 (version 25.0; SPSS Inc., Chicago, IL, USA) were used for statistical analysis.

## 3. Results

The PD mouse model was induced by injecting MPTP-HCL (20 mg/kg) three times every 2 h, and experimental treatment was conducted after 2 h (Figure 1). To examine motor ability, the rotarod test was performed in the control, negative control (NC), *Serping1* siRNA injection (SER1), and *N*-acetylcysteine (NAC) groups (control, only PBS was injected; NC, transfection reagent with PBS was injected in MPTP-treated mice; SER1, *Serping1* siRNA and transfection reagent with PBS were injected in MPTP-treated mice; NAC, NAC with PBS was injected in MPTP-treated mice). Although motor ability significantly decreased in the NC and NAC groups (*p* < 0.05), that of the SER1 group was maintained at the level of the control group (Figure 2B).

**Figure 3:**
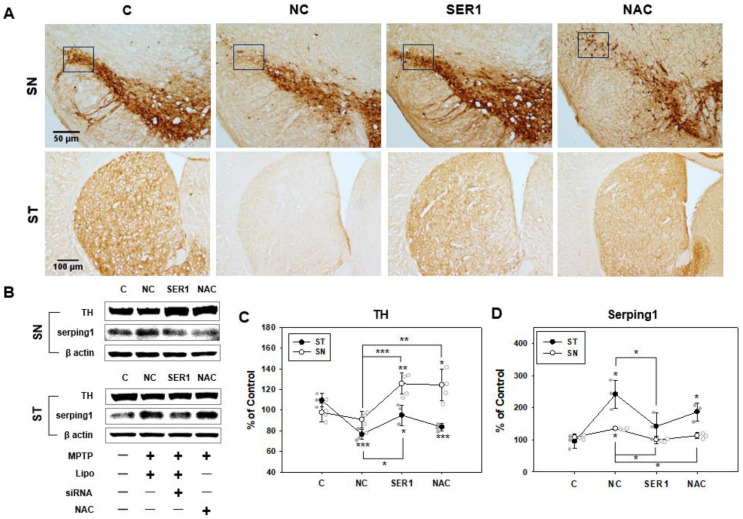
Tyrosine hydroxylase (TH) and *Serping1* in striatum (ST) and substantia nigra (SN). (**A**) Immunohistochemical analysis of the changes in TH levels in ST and SN were observed in the C, NC, SER1, and NAC groups (C, only PBS was injected; NC, transfection reagent with PBS was injected in 1-methyl-4-phenyl-1,2,3,6-tetrahydropyridine (MPTP)-treated mice; SER1, *Serping1* siRNA and transfection reagent with PBS were injected in MPTP-treated mice; NAC, NAC with PBS was injected in MPTP-treated mice). Decreasing TH levels in the substantia nigra pars compacta (SNpc) shown in the rectangular box were repressed in the SER1 and NAC groups. Decreasing TH levels in the ST region were inhibited in the SER1 and NAC groups; upper and lower panels: scale bars = 50 and 100 μm, respectively. (**B**) Immunoblotting analysis of TH and *Serping1* in each group. While TH levels decreased in SN and ST of the NC group, decreasing TH levels were inhibited in the SER1 group more than in the NAC group. *Serping1* levels increased in SN and ST of the NC group, but increasing *Serping1* levels were inhibited significantly in SN and ST of the SER1 group. (**C**) The blotting analysis of TH in the SN and ST regions is described in a graph (ST, *n* = 3, F(3,8) = 14.076, ANOVA = 0.001; SN, *n* = 5, F(3,16) = 13.068, ANOVA < 0.001). (**D**) The blotting analysis of *Serping1* in the SN and ST regions is described in a graph (ST, *n* = 3, F(3,8) = 9.495, ANOVA = 0.005; SN, *n* = 3, F(3,8) = 8.746, ANOVA = 0.007); * *p* < 0.05, ** *p* < 0.005, *** *p* < 0.0005.

Pathological signals in PD are represented by decreases in TH in the SN and ST due to dopaminergic cell destruction in the SN. In immunohistochemical analysis, changes in TH levels in the SN and striatum regions were observed in the control, NC, SER1, and NAC groups to confirm the pathological alterations in differently treated groups (Figure 3). The TH level decreased in dopaminergic cells in the substantia nigra pars compacta (SNpc) and striatum regions in the NC group (rectangular box in Figure 3A). However, the decrease in TH levels due to MPTP treatment and the destruction of dopaminergic cells were inhibited by the injection of *Serping1* siRNA and antioxidant NAC in the SER1 and NAC groups, respectively (Figure 3A).

The expression levels of TH and *Serping1*, the pathological indicator of PD, were investigated in the SN and striatum regions in each group using immunoblotting (Figure 3B). Although TH expression levels significantly decreased in the striatum regions of the NC group due to MPTP treatment, significantly increased TH levels were observed in the striatum regions of the SER1 group, as well as in the NAC group treated with antioxidant NAC (Figure 3B,C). These results indicate that treated *Serping1* siRNA had a positive effect on striatum regions regarding TH levels. Significantly decreased *Serping1* expression levels were also observed in the SN and striatum of the brain in the SER1 group (*p* < 0.05, Figure 3B,D).

**Figure 4:**
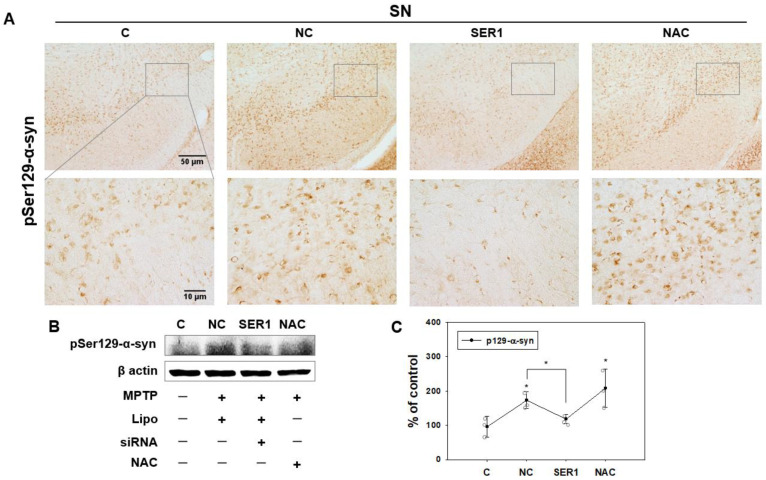
Phosphorylated (Ser 129)-α-synuclein (pSer129-α-syn) in SN. (**A**) Immunohistochemical analysis of pSer129-α-syn in SNpc in each group. pSer129-α-syn levels increased in the SNpc region (rectangular box) of the NC and NAC groups. However, pSer129-α-syn levels decreased in SNpc of the SER1 group; upper and lower panels: scale bars = 50 and 10 μm, respectively. (**B**) Immunoblotting analysis of pSer129-α-syn in the SN region in each group. pSer129-α-syn levels increased in the SN region of the NC and NAC groups, but did not increase in the SER1 group. (**C**) Immunoblotting analysis of p-a-syn in SN of each group is shown in a graph (*n* = 3, F(3,8) = 6.525, ANOVA = 0.015); * *p* < 0.05.

Immunohistochemistry analysis of pSer129-α-syn, a potential crucial factor in PD, showed that pSer129-α-syn levels increased in the SN and SNpc (rectangular box in Figure 4A) in the NC and NAC groups (Figure 4A). However, pSer129-α-syn levels decreased in the SN and SNpc in the SER1 group. These changes were also observed in the immunoblot analysis of SN (Figure 4B). The expression level of pSer129-α-syn was significantly lower in the SER1 group than in the NC and NAC groups (*p* < 0.05, Figure 4C). *Serping1* siRNA was found to have a positive effect on inhibiting the increased phosphorylation of α-syn in SN.

**Figure 5:**
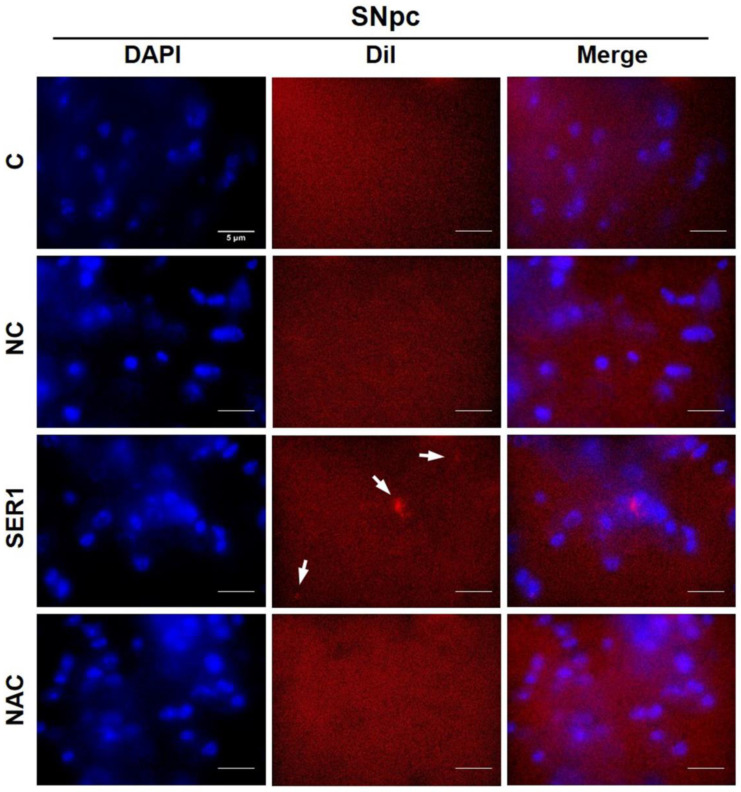
Observation of dialkyl indocarbocyanine (DiI) in SNpc. Seven days after therapeutic materials, including DiI, were treated, SN was observed to examine whether the therapeutic materials reached SN or not. DiI materials were observed in the SER1 group. Cell nuclei of each group dyed with 4′,6-diamidino-2-phenylindole (DAPI) were presented in each group. Panels observing cell nuclei and DiI were merged. Scale bar = 5 μm.

To examine whether the injected materials reached the dopaminergic cells of the SN, DiI was included in the treatment materials in each group. The SN area was observed post treatment 7 days later, and the presence of injected DiI was detected in the SER1 group (Figure 5). Therefore, it can be inferred that injected materials reached the SN area in the SER1 group.

**Figure 6:**
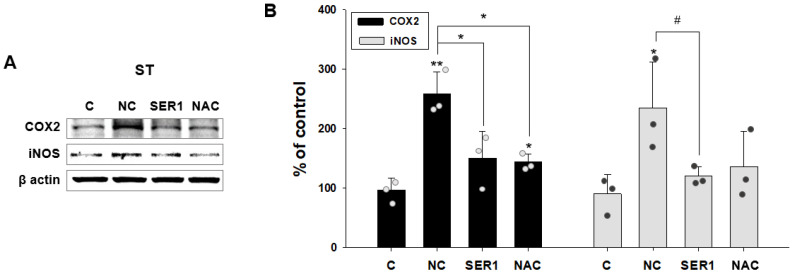
Changes in cyclooxygenase-2 (COX2) and inducible nitric oxide synthase (iNOS) levels in the striatum by injecting *Serping1* siRNA. (**A**) Immunoblot results of COX2 and iNOS in the striatum of C, NC, SER1, and NAC groups. (**B**) The immunoblot of COX2 and iNOS is shown in a graph. COX2 and iNOS levels increased significantly in the NC group. However, COX2 levels decreased significantly in the SER1 and NAC groups, and iNOS levels also decreased in the SER1 and NAC groups; (COX2, *n* = 3, F(3,8) = 14.270, ANOVA = 0.001; iNOS, *n* = 3, F(3,8) = 4.366, ANOVA = 0.042) means ± standard errors, * *p* < 0.05, ** *p* < 0.005, # *p* = 0.067.

Meanwhile, the expression levels of the factors COX2 and iNOS, and their propensity to create an inflammatory reaction, were verified in the striatum regions in each group (Figure 6A). Although COX2 and iNOS expression levels increased significantly in the NC group because of inflammation, inflammatory factor levels decreased in the SER1 and NAC groups. In particular, the COX2 expression level was significantly decreased in the SER1 and NAC groups (*p* < 0.05, Figure 6A,B).

## 4. Discussion

In this study, a PD mouse model induced by MPTP was created, and four groups (control, NC, SER1, and NAC) were compared according to treatment materials. NAC is emerging as a therapeutic agent for a variety of vascular and non-vascular neuropathies and neurodegenerative diseases, including PD [17]. NAC was used as a positive control to compare the positive effect of *Serping1* siRNA on PD as a potential therapeutic material. NAC was included to provide a baseline for comparison when administering the potential therapeutics. However, NAC and *Serping1* siRNA differ substantially in their mechanisms of action, as NAC exerts broad antioxidant and systemic effects, whereas *Serping1* siRNA specifically targets the inflammatory–complement pathway. Therefore, NAC was used as a mechanistically distinct comparator in this study.

DiI was observed in the SN to examine whether the injected materials reached the SN. DiI was detected in the SER1 group (Figure 5). DiI is a widely used lipophilic fluorophore that intercalates into lipid bilayers, labeling membranes with fluorescence. Similarly, Lipofectamine acts as a delivery vehicle that encapsulates genetic material within a lipid bilayer (forming a lipoplex) to facilitate intracellular transport. Due to its lipophilic nature, DiI molecules are readily incorporated into the lipid structure of the Lipofectamine complex. However, over time, the dye may diffuse toward the cell membrane upon contact with the lipoplex. This suggests that DiI-based visualization provides an indirect measurement, as it primarily tracks the Lipofectamine carrier rather than the siRNA itself. Therefore, it could be inferred that injected materials reached the SN area in the SER1 group.

*Serping1* levels in the SN increased in the NC group, but decreased in the SER1 and NAC groups (Figure 3B,D). These results suggest that *Serping1* siRNA in SN reduced the *Serping1* expression level. The inherent complexity of in vivo studies can make it challenging to definitively categorize whether the *Serping1* upregulation is causative, compensatory, or epiphenomenal in the MPTP-induced PD model. However, several findings from earlier studies [6] point toward a pathogenic contribution rather than a transient compensatory response. Had the elevation of *Serping1* been a temporary compensatory mechanism, its expression would have diminished in the 4-week MPTP-induced PD model. In contrast, the previous report has confirmed that *Serping1* levels remained elevated in the chronic stage. Furthermore, the observation that the knockdown of *Serping1* resulted in a reduction in α-syn and an increase in TH provides functional support for its role in disease progression. Therefore, the upregulation of *Serping1* appears to be more than a mere epiphenomenon. Nevertheless, it remains elusive whether the upregulation of *Serping1* in PD is a consequence of PD pathology or actively contributes to its pathogenesis.

In the SN region, the NC group showed increased pSer129-α-syn levels as a pathological symptom of PD (Figure 4), whereas the SER1 group showed a comparatively greater decrease in pSer129-α-syn levels. Thus, it can be deduced that *Serping1* siRNA affects pSer129-α-syn formation by inhibiting it, where *Serping1* may be related to the phosphorylation (Ser129) of α-syn as a serine or cysteine peptidase inhibitor. The reduction in pSer129 α-syn following *Serping1* knockdown is likely secondary to enhanced proteolytic clearance. Loss of Serping1, a major serine protease inhibitor, may disinhibit enzymes such as plasmin or neurosin (KLK6) [18,19,20,21], leading to accelerated degradation of total α-syn. As phosphorylation depends on substrate availability, depletion of α-syn would reduce pSer129 levels indirectly, without requiring direct modulation of kinase/phosphatase activity. However, the data in this study do not sufficiently support this proposed mechanism or a direct regulatory role of *Serping1* in α-syn phosphorylation, and further work will be needed to fully elucidate the precise molecular mechanisms of *Serping1* action.

*Serping1* regulates complement activation by inhibiting activated C1r and C1s of the first complement component, and Serpins have also been implicated in inducing cell death [5,22,23]. We propose the following hypothesis: *Serping1* siRNA could inhibit the induction of cell death by increasing *serping1* expression in dopaminergic cells.

Furthermore, the expression levels of factors related to inflammatory reactions, COX2 and iNOS, decreased in the SER1 and NAC groups in the striatum region (Figure 6). As *Serping1* is involved in inflammation and the inflammation spreads from striatum to SN in PD [1,24], it is thought that it could be critical to decrease the inflammation reaction in the striatum. Consequently, *Serping1* siRNA and NAC may have therapeutic potential for PD.

siRNAs are attracting attention as potential and promising therapeutics because the first RNA interference therapeutic was approved in 2018. However, efficient delivery systems are still being actively researched. A limitation of this study is that it did not overcome the efficient delivery and *Serping1* siRNA could affect the brain indirectly. While intraperitoneal siRNA delivery via Lipofectamine was employed as a proof-of-concept approach to evaluate the functional role of *Serping1*, this method allowed for the observation of improvements in the MPTP-induced PD model. However, translating these findings will require more sophisticated delivery vehicles, such as lipid nanoparticles or viral vectors, which are currently being optimized for central nervous system targeting in clinical trials [25,26,27]. *Serping1*, a member of the serine protease inhibitor family G1, modulates complement component C1. In addition, this protein participates in regulating both the kallikrein–kinin pathway and plasminogen activation, thereby playing a role in inflammatory processes [28]. In the kinin system, bradykinin receptor 1 activates endothelial nitric oxide synthase and induces local edema formation and fluid extravasation [29,30]. Furthermore, stimulation of bradykinin receptor 1 led to reduced occludin levels at tight junctions, accompanied by enhanced vascular permeability and disruption of blood–brain barrier integrity [31]. Based on these reports, *Serping1* siRNA may have exerted an indirect effect on PD by mitigating neuroinflammation or through vascular integrity loss. The findings of this study do not suggest that *Serping1* upregulation is a primary pathogenic mechanism of PD. However, these results are meaningful in that *serping1* may be part of various alterations in signaling pathways associated with PD.

A primary limitation of this study is the lack of definitive validation for the *Serping1* antibody using genetic knockout controls. The presence of non-specific bands on our immunoblots means that the quantification of *Serping1*, and the conclusions derived from it, should be considered preliminary. We acknowledge as a limitation that different vehicles were used for NAC and siRNA delivery. As a hydrophilic small molecule, NAC readily dissolves in aqueous solutions and is administered in its free form. This contrasts with the siRNA used in this study, which requires encapsulation into lipid-based lipoplexes via Lipofectamine to facilitate cellular entry. This discrepancy in physicochemical properties and delivery methods represents a limitation, as the pharmacokinetics of a hydrophilic compound may differ significantly from those of a lipid-based delivery system. Future studies should include additional controls to more definitively isolate treatment effects from any potential vehicle contribution and non-targeting (scrambled) siRNA. The *Serping1* siRNA treatment group showed beneficial effects on PD, despite the delivery of a small amount of *Serping1* siRNA. Therefore, *Serping1* siRNA could represent a potential candidate for PD therapy, pending further validation.

Nonetheless, several challenges remain to be addressed. The current study lacks a quantitative assessment of siRNA levels in the brain, a demonstration of cell-type specificity, and controls for potential systemic or vascular effects. Therefore, to better understand the effects of *Serping1* siRNA on PD, studies providing direct and quantitative measurements of *Serping1* siRNA delivery to the brain are needed. Based on reports suggesting an association between *Serping1* and neuroinflammation, examining cell-specific colocalization using glial markers and observing changes in glial cells represent an alternative and promising research direction. Furthermore, given the established roles of *Serping1* in vascular integrity and complement pathways, the neuroprotection observed in the MPTP model may be partially attributed to secondary peripheral or vascular stabilization rather than exclusive neuronal modulation. The potential contribution of systemic complement inhibition and blood–brain barrier maintenance requires further rigorous investigation to delineate the precise mechanism of action. Also, although a larger sample size would have provided greater statistical power and broader generalizability, the current study is limited by its relatively small sample size. Consequently, the findings should be interpreted with caution, and further validation in a larger cohort is warranted. In addition, as this study does not provide detailed information regarding various dosing, toxicity, or systemic effects, further investigation is needed to address these aspects in future studies.

## 5. Conclusions

The SER1 group showed an almost normal motor status like the control group in the motor ability test. At the TH level, the SER1 group also showed an almost normal TH expression status in the SN and striatum. It can be deduced that injected materials reached the SN region by observing DiI, and *Serping1* levels in the SN increased in the NC group but decreased in the SER1 group owing to *Serping1* siRNA. Regarding pSer129-α-syn levels in the SN region, *Serping1* siRNA had a positive effect on PD by inhibiting pSer129-α-syn formation. In addition, considering that it was anticipated that NAC would reduce the inflammatory reaction as an antioxidant, our results showed that *Serping1* siRNA also has a positive effect on inflammation in the striatum as NAC, in that COX2 and iNOS levels decreased in the SER1 group. Therefore, both *Serping1* siRNA and NAC demonstrated a beneficial effect on the pathological changes associated with PD in our study. If an efficient delivery system is developed, it would be meaningful to continue research on *Serping1* siRNA as a potential therapeutic strategy.

## Data Availability

The original contributions presented in this study are included in the article. Further inquiries can be directed to the corresponding author(s).
